# Evaluating the feasibility and exploring the efficacy of an emotion-based approach-avoidance modification training (eAAMT) in the context of perceived stress in an adult sample — protocol of a parallel randomized controlled pilot study

**DOI:** 10.1186/s40814-023-01386-z

**Published:** 2023-09-07

**Authors:** Marie Keinert, Bjoern M. Eskofier, Björn W. Schuller, Stephanie Böhme, Matthias Berking

**Affiliations:** 1 Department of Clinical Psychology and Psychotherapy, Friedrich-Alexander-Universität Erlangen-Nürnberg (FAU), Erlangen, 91052 Germany; 2 Machine Learning and Data Analytics Lab, Department Artificial Intelligence in Biomedical Engineering, Friedrich-Alexander-Universität Erlangen-Nürnberg (FAU), Erlangen, Germany; 3 Chair of Embedded Intelligence for Health Care and Wellbeing, University of Augsburg, Augsburg, Germany; 4 GLAM, Imperial College London, London, UK; 5 Chair for Clinical Psychology and Psychotherapy, Department of Psychology, Technische Universität Chemnitz, Chemnitz, Germany

**Keywords:** Perceived stress, Dysfunctional beliefs, Approach-avoidance modification, Emotion, Smartphone-based intervention, Parallel randomized controlled pilot trial

## Abstract

**Background:**

Stress levels and thus the risk of developing related physical and mental health conditions are rising worldwide. Dysfunctional beliefs contribute to the development of stress. Potentially, such beliefs can be modified with approach-avoidance modification trainings (AAMT). As previous research indicates that effects of AAMTs are small, there is a need for innovative ways of increasing the efficacy of these interventions. For this purpose, we aim to evaluate the feasibility of the intervention and study design and explore the efficacy of an innovative emotion-based AAMT version (eAAMT) that uses the display of emotions to move stress-inducing beliefs away from and draw stress-reducing beliefs towards oneself.

**Methods:**

We will conduct a parallel randomized controlled pilot study at the Friedrich-Alexander-Universität Erlangen-Nürnberg, Germany. Individuals with elevated stress levels will be randomized to one of eight study conditions (*n* = 10 per condition) — one of six variants of the eAAMT, an active control intervention (swipe-based AAMT), or an inactive control condition. Participants in the intervention groups will engage in four sessions of 20–30 min (e)AAMT training on consecutive days. Participants in the inactive control condition will complete the assessments via an online tool. Non-blinded assessments will be taken directly before and after the training and 1 week after training completion. The primary outcome will be perceived stress. Secondary outcomes will be dysfunctional beliefs, symptoms of depression, emotion regulation skills, and physiological stress measures. We will compute effect sizes and conduct mixed ANOVAs to explore differences in change in outcomes between the eAAMT and control conditions.

**Discussion:**

The study will provide valuable information to improve the intervention and study design. Moreover, if shown to be effective, the approach can be used as an automated smartphone-based intervention. Future research needs to identify target groups benefitting from this intervention utilized either as stand-alone treatment or an add-on intervention that is combined with other evidence-based treatments.

**Trial registration:**

The trial has been registered in the German Clinical Trials Register (Deutsches Register Klinischer Studien; DRKS00023007; September 7, 2020).

## Background

The World Health Organization has recently referred to stress as “the health epidemic of the twenty-first century” [1]. Reasons for this assessment included data indicating that stress has significantly increased during the past decades [2] and hence increasingly burdens individuals with significant physical (e.g., [3]) and mental (e.g., [4–6]) health risks. This also leads to higher direct and indirect stress-related costs for societies [7]. On top of this trend, the COVID-19 pandemic has increased stress burden even further for many individuals [8, 9]. Thus, there is a need for effective and accessible interventions fostering effective stress management.

Ideally, such interventions should be grounded on validated theories on stress. According to the transactional stress model proposed by Lazarus and Folkman [10], stress results from a situation that is interpreted as significant threat to the attainment of salient goals and from a subsequent assessment that appraises presently available resources as insufficient to cope with the eminent threat. Stress is reflected in the activation of physiological stress systems. It activates the hypothalamic–pituitary–adrenal axis leading to the release of stress hormones like cortisol [11] and the autonomic nervous system which results in the release of salivary alpha-amylase [12] as well as changes in heart rate variability and breathing rate [13, 14]. Moreover, stress is associated with a subjective experience of situations to be stressful, also referred to as perceived stress [15]. Since the cognitive act of appraising both the situation and one’s coping resources is central to the emergence of stress, it constitutes an important target in interventions aiming to facilitate successful coping with stress [16, 17]. Such appraisals are influenced by objective characteristics of the situation (e.g., controllability, intensity, duration) as well as by commitments and beliefs [10]. For example, if a person believes that they must “be perfect,” they are more likely to appraise a certain achievement as insufficient. Consequently, they will experience stress, as perceived insufficient goal attainment will threaten their self-esteem [18, 19].

Empirically, previous studies found dysfunctional beliefs to be related to perceived stress [20] and to moderate the association between stressful events and dysphoria/depression [21–24]. Therefore, many stress management trainings include interventions that intend to invalidate stress-related beliefs (e.g., “If I am not good at what I do, I am not a worthy person”) while promoting beliefs likely to reduce stress (e.g., “My value as a person does not depend on work-related achievements,” e.g., [25, 26]).

In common face-to-face stress management trainings, stress-related beliefs are usually targeted with cognitive restructuring, where the therapist guides participants to the insight that stress-related beliefs are neither valid nor healthy and helps them to complement or replace the beliefs with more valid and healthier ones [27]. As this approach is very much personalized, it requires direct interaction with a well-trained therapist. Thus, it is comparatively expensive and very difficult to integrate in easy-to-disseminate computerized interventions. This is problematic insofar as computerized interventions can easily be delivered through the Internet at relatively low prices and, hence, hold strong potential for improving healthcare services worldwide. Similar to face-to-face trainings, current online-based stress management trainings introduce participants to the stress model [10] and provide exercises to modify stress-related beliefs (e.g., [28–31]), the difference being that they are conducted without the support of a therapist. One disadvantage of this kind of digital self-help is that it requires more self-motivation and self-management skills than available to many individuals suffering from high stress burden. In addition, this approach sorely lacks reinforcing experiences which in face-to-face-treatment are provided by the therapists. This, in our clinical experience, often leads to underutilization of the intervention. For this reason, there is a need for ways of digitally delivering cognitive stress management techniques in a more engaging, more gamified way.

Theoretically, such a new approach can be based on cognitive bias modification (CBM). CBM interventions are techniques of cognitive behavioral therapy aiming to change cognitive biases in mental disorders on the level of automatic processing [32]. The approach-avoidance modification training (AAMT), for example, systematically targets approach-avoidance biases typically found in various mental disorders [33], thereby changing the evaluation of and behavioral reactions to disorder-specific stimuli. AAMTs are based on the assumption that repeatedly approaching or avoiding a stimulus influences its affective evaluation [34]. That is, avoiding a stimulus leads to a more negative and approaching one to a more positive evaluation. According to van Dessel and colleagues’ inferential account [35], these findings can be explained by a (nonconscious) inferential process where the evaluation of a certain action is transferred to the stimulus it relates to. Hence, if a certain action is “coded” as positive, the stimulus this action refers to will be evaluated as positive, which in turn will facilitate approach behavior towards this stimulus in the future (e.g., “I find myself approaching this stimulus repeatedly, so it must be positive (and shall therefore be approached in the future)”).

Originally developed in the field of anxiety [36], the standard AAMT is administered on a computer. Participants are instructed to respond to a certain stimulus by pushing or pulling a joystick. In addition to the physical approach/avoidance movements, a virtual distance change is implemented: pulling the joystick enlarges the stimulus to simulate approach while pushing it shrinks the stimulus to simulate avoidance. Variants of this method include using mouse movements (e.g., [37]) or moving a manikin towards or away from a stimulus through key presses (e.g., [38]). Recent technological developments allow an adaptation of the paradigm for use on the smartphone, where participants are asked to swipe the stimuli on a touchscreen instead of using a joystick [39], greatly facilitating the dissemination of the intervention compared to desktop-based AAMTs (e.g., [40, 41]).

Several studies applied AAMTs as a(n adjunctive) treatment for mental health problems. By far, the most have been conducted in the field of substance-related disorders. In alcohol use disorder, AAMTs reduced both approach biases towards substance-related stimuli (*d* = 0.51) and relapse rates (*d* = 0.61; e.g., [42–44]). Evidence is less clear in nicotine use disorder, anxiety disorders, and eating disorders [33]. Some studies did find effects on approach-avoidance biases and/or symptomatology (e.g., [45–47]), such as approach bias reduction towards food stimuli in bulimic eating disorder psychopathology (*d* = 0.73), an increase of social approach behavior in individuals with social anxiety (*d* = 0.79), and a reduction of cigarette consumption (*d* = 0.35), and dependence (*d* = 0.41), whereas other studies did not (e.g., [48–50]). Similarly, an approach training towards positive stimuli did not affect mood in dysphoric individuals [51], whereas studies that combined a short counselling session with an AAMT targeting depressogenic beliefs have been shown to significantly reduce depressive symptoms (*d* = 1.02) [41]. Similar combinations of short counselling sessions and subsequent AAMT proved to be effective in the treatment of body dissatisfaction [40], procrastination [52], and alexithymia [53].

Despite these encouraging findings, there are only two studies employing an AAMT in the stress context. Here, Ferrari and colleagues [54] investigated the effects of an AAMT on stress reactivity to a stress-eliciting task. In the AAMT, participants trained to approach positive pictures and/or avoid negative pictures originating from the International Affective Picture System [55]. Although approaching positive pictures and avoiding negative pictures did increase approach tendencies towards positive pictures, there were no effects on physiological stress measures or perceived stress. Becker and colleagues [51], on the other hand, found reduced stress reactivity in dysphoric individuals after approaching positive and avoiding negative pictures.

As outlined above, AAMTs have been investigated as promising treatments for various forms of psychopathology, with, albeit, much room for further improving their efficacy in particular when used as standalone interventions. Most prominently, based on the inferential account [35], it can be argued that the finger or wrist movements performed in previous AAMTs do not represent valence with the necessary clarity/intensity. Thus, the efficacy of AAMTs might be increased by utilizing responses that clearly represent salient desired or undesired states. Such responses should carry strong positive or negative valence, which will, arguably, be transferred to the stimuli used in the AAMT and, consequently, foster approach or avoidance, respectively.

Emotions constitute an auspicious candidate for such responses. Emotions can be conceptualized as complex information processing schemes [56] that are cued whenever emotion-specific patterns are detected in the information provided directly from sensory input (i.e., “fast path” towards emotion elicitation) and/or the cognitive subsystems processing this input (i.e., “slow path” towards emotion elicitation). When cued, emotions initiate a coordinated set of cognitive, somatic, motivational, and behavioral responses aiming to minimize perceived discrepancies between desired and perceived states [57]. As undesired (i.e., “negative”) emotions usually imply that the present state is unsatisfactory and as all desired (i.e., “positive”) emotions usually imply that the present state is satisfactory, emotions carry strong valence signals. Thus, it can be hypothesized that moving AAMT stimuli with the help of emotional responses will transfer stronger positive and negative valence to the respective stimuli than with mere finger or wrist movements. Therefore, an emotion-based AAMT (eAAMT) should have stronger effects on behavior than the standard swipe AAMT. While there are several emotions with a negative (i.e., anxiety, anger, sadness, and disgust) or positive valence [58], these emotions differ in the specific appraisal they elicit and the behavioral tendencies they cue [59], which will be inferred to the respective stimulus. Hence, they might differ in their effectiveness when used within an eAAMT.

In line with embodiment and facial feedback theories [60], we assume that the mere display of emotions (in the sense of play-acting) in different modalities might suffice to cue such an emotional response. There is evidence that purposefully displaying facial, gestural, and verbal expressions of emotions can elicit emotional responses. For example, in a recent meta-analysis, Coles and colleagues [61] found, albeit small, effects of emotional facial expressions on emotional experience. Other studies found similar effects for emotional expressions in body and voice (e.g., [62–65]). But even if a person does not consciously experience the respective emotion, its display will likely carry a significantly stronger positive or negative valence than the simple finger or hand movements performed in swipe or joystick AAMTs.

To investigate these assumptions, we developed an eAAMT in which participants display positively and negatively valenced emotions to approach or avoid stimuli. Given the importance of stress-related problems and the lack of studies on AAMT-based interventions against stress, our eAAMT targets stress. As stress-related beliefs play an important role in its emergence, stimuli will consist of statements representing stress-reducing and stress-enhancing beliefs. Because of the need for accessible and easy-to-disseminate interventions against stress, we developed the eAAMT as a (guided) smartphone-based intervention.

### Purpose of the study

Before conducting a large RCT on the eAAMT, we aim to test intervention and study procedures and estimate preliminary effect sizes on clinical outcomes. For this purpose, we will run a pilot study with the following aims: (a) evaluate the feasibility of the intervention and study design and (b) explore its efficacy in influencing clinical outcomes when compared to an inactive control condition and a swipe-based AAMT (swipe control condition). Additional exploratory questions are whether the specific emotions anxiety, anger, sadness, and disgust differ with respect to their effectiveness in eAAMTs and whether deviating from the common 50:50 ratio of approach vs. avoidance responses (e.g., [35, 47]) in favor of a 1:4 bias towards approach responses would lead to stronger effects on the reduction of perceived stress.

## Methods

### Design

**Fig. 1:**
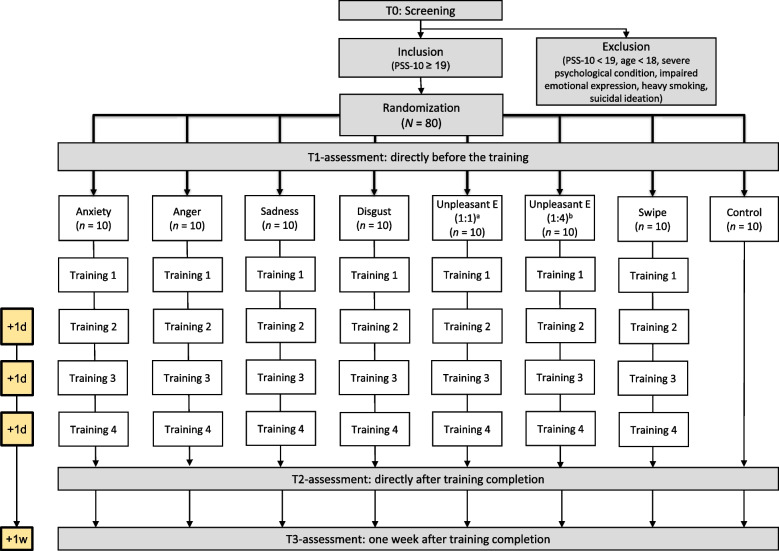
Study procedure. Note: PSS-10, Perceived Stress Scale 10; E, emotion; d, day; w, week. ^a^All unpleasant emotions (i.e., disgust, anxiety, sadness, and anger) are used to avoid dysfunctional beliefs. The approach-avoidance response ratio is 1:1. ^b^All unpleasant emotions are used to avoid dysfunctional beliefs, but the approach-avoidance response ratio is 4:1

We will conduct a parallel eight-armed randomized controlled pilot study (see Fig. 1 for the study flow and supplemental materials for the spirit checklist of the trial). The study will be conducted at the Department of Clinical Psychology and Psychotherapy of the Friedrich-Alexander-Universität Erlangen-Nürnberg. In the eAAMT and swipe control groups, participants will receive four sessions à 20–30 min of training on four consecutive days, while the inactive control group will not receive any training. Participants will be randomly assigned to one of the eight study groups with a 1:1 allocation. A postdoctoral researcher not otherwise involved in the study will generate a randomization list using random numbers in Microsoft Excel. A block randomization will be performed with a block size of eight. The study is registered in the German Clinical Trials Register (Deutsches Register Klinischer Studien; DRKS00023007).

### Participants

Inclusion criteria for participation in the pilot study will be the following: (1) heightened levels of perceived stress as indicated by a score of 19 or above on the Perceived Stress Scale-10 (PSS-10; German version: [66]), (2) at least 18 years of age, and (3) ability and willingness to provide informed consent. Following procedures suggested by Heber and colleagues [67], the PSS cut-off score is set at one standard deviation above the PSS mean score in the validation study of the German version of the PSS-10 [66]. Exclusion criteria will be as follows: (1) the presence of a severe psychological condition (i.e., psychosis; based on self-report), (2) physical conditions impairing the expression of emotion (e.g., facial paralysis), (3) heavy smoking (i.e., more than 10 cigarettes/day due to possible confounding influences on cortisol measures), and (4) suicidal ideation. We expect a medium to large effect size based on previous smartphone-based AAMT studies (e.g., [52, 53]). Therefore, we will include *n* = 10 participants per study group (i.e., *N* = 80 in total) as suggested by Bell and colleagues [68] for pilot studies with expected medium to large effect sizes. To complete the intervention per protocol, participants should attend at least three of the four training sessions. To ensure even distribution of subsample sizes across study conditions, we will change randomization block size in case of treatment dropouts. Number of and reasons for dropping out will be recorded and reported for each condition.

### Interventions

#### Experimental interventions

The experimental interventions will consist of six variations of the eAAMT (see below for details) delivered by a smartphone app. We decided to include these variations to be able to compare the utility of different negative emotions (i.e., anger, anxiety, disgust, and sadness) as avoidance responses. Moreover, we want to investigate whether the ratio of approach to avoidance responses has an influence on the effects of the training. Therefore, we will include one training condition using a ratio of 4:1 of approach vs. avoidance responses. The intervention aims to reduce perceived stress by modifying stress-related beliefs; therefore, the AAMT stimuli will consist of statements representing dysfunctional (e.g., “If I fail partly, it is as bad as a complete failure”) and functional (e.g., “I am allowed to make mistakes”) beliefs.

**Fig. 2:**
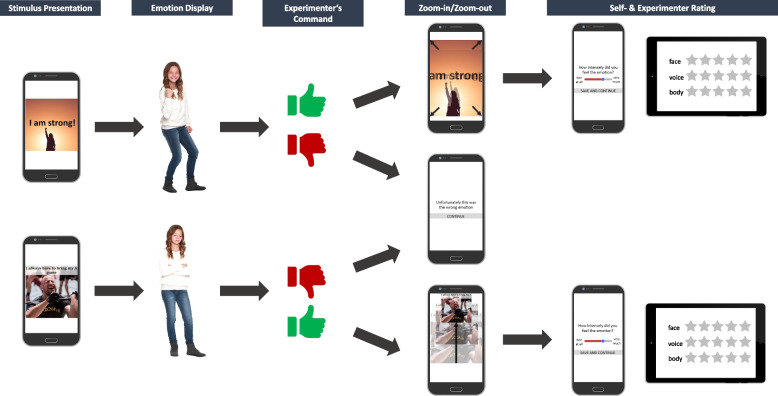
Exemplary trial flow

The pool of 78 statements in total (30 stress-enhancing and 48 stress-reducing beliefs — for the eAAMT-unpleasant emotions 1:4 condition, more stress-reducing beliefs are needed; see below) will consist of items from the Dysfunctional Attitude Scale (DAS) [69] complemented by items proposed by a team of three experts in the development of stress management interventions (the senior author and two licensed psychotherapists and stress experts working in our group). To make the intervention more appealing, the statements will be presented on the screen with pictures relating to the statements (see Fig. 2 for examples).

The study sessions will be held in a lab at the Department of Clinical Psychology and Psychotherapy of the Friedrich-Alexander-Universität Erlangen-Nürnberg. Each participant will participate in four 20–30-min training sessions on consecutive days. Participants will be provided with a smartphone for the training. The sessions will be conducted by undergraduate and graduate students (experimenters) who will be trained and supervised by a postdoctoral researcher. In addition, they will be guided by a manual outlining the procedure and instructions of the experiment.

The experimenters will explain the aim of the training and its implementation. In addition, the app will provide participants with short written instructions for the training which will be complemented with short video clips. Each training session will consist of 60 trials where the stimuli are presented in random order. With the exception of one condition (see below), the approach-avoidance response ratio will be 50:50. Upon presentation of a stimulus, participants will decide whether they should pull the respective belief towards themselves or push it away (i.e., whether it is stress enhancing or stress reducing). Participants will draw stress-reducing beliefs towards themselves by displaying one of twelve positive emotions. The training sessions will be divided into three blocks (20 trials each) with participants being instructed to change the emotional response after each block. The emotions will always be displayed in the same order (day 1: joy, relaxation, and love; day 2: excitement, tranquility, and gratitude; day 3: happiness, resolve, and content; day 4: courage, confidence, and pride). Stress-enhancing beliefs will be pushed away by displaying different negative emotions, namely anxiety, anger, disgust, and sadness. The instructive video clips will show an actor who carries out the display of each emotion. The video clips will address the following four components: (1) facial expression of the emotion, (2) a verbal statement consistent with the emotion, (3) the intonation of the verbal statement, and (4) a body gesture consistent with the emotion. When the correct emotion is displayed, the stimuli will either zoom in (approach) or out (avoidance), and a thumbs-up picture will be presented on the smartphone screen to systematically reinforce possible effects of the training. When the stimulus is not processed correctly, the sentence “Unfortunately, this was the wrong emotion” will appear on the screen. Figure 2 shows an exemplary trial flow. In all eAAMT conditions, an investigator will observe participants’ emotion display through a live stream of a video camera from behind a partition wall and remote control the app with a tablet. Smartphone and tablet are connected using Google’s Nearby Application Programming Interface (https://developers.google.com/nearby/), which automatically establishes either a Bluetooth or a Wi-Fi connection between the two devices.

The six variations of the eAAMT are described in the following:

- *eAAMT anxiety*: In the eAAMT-anxiety condition, participants will be asked to push stress-enhancing beliefs away from themselves by displaying anxiety. Anxiety conveys the information that a stimulus is dangerous and cues avoidance tendencies [57, 70]. It might thus be helpful in increasing the avoidance motivation towards stress-enhancing beliefs. As instructed by the actor shown in the introductory video, participants are asked to express anxiety by a fearful facial expression and heavy breathing, by withdrawing the body, and saying “It is dangerous to think like that, I will protect myself from that!”.
- *eAAMT anger*: In the eAAMT-anger condition, participants will be asked to push stress-enhancing beliefs away from themselves by displaying anger. Anger is activated when someone or something is hindering a person to attain a personally relevant goal and when apologetic circumstances are not seen [57]. While anxiety cues avoidance tendencies, anger facilitates fighting for one’s goals [57]. Participants are instructed to display anger by an angry facial expression, a threatening clench of the fist, hitting the table, and saying “Such a stupid belief, I will not listen to its advice!”.
- *eAAMT disgust*: In the eAAMT-disgust condition, participants will be asked to push stress-enhancing beliefs away from themselves by displaying disgust. The evolutionary function of disgust was to detect contaminated/poisonous food or disease threat [71]. Therefore, it triggers strong aversion and is associated with avoidance tendencies [58, 70]. Disgust will be expressed by a disgusted facial expression, turning away one’s head, and saying “Ugh, this is disgusting, I will stay away from that!”.
- *eAAMT sadness*: In the eAAMT-sadness condition, participants will be asked to push stress-enhancing beliefs away from themselves by displaying sadness. Sadness provides the information that current goals are not attainable and cues a letting go of these goals, which eventually facilitates committing to other (more attainable) goals [57]. When used within the eAAMT, sadness might help participants to regret that they have developed dysfunctional beliefs and let go of them. Participants are instructed to display sadness by a sad facial expression and deep breath, tilting the head forward, and saying “I will let go of this belief!”.

Furthermore, two conditions will be implemented to allow studying the role of the approach-avoidance-response ratio.

- *eAAMT-unpleasant emotions 1:1*: In the eAAMT-unpleasant emotions 1:1 condition, participants will display a different unpleasant emotion to push stress-enhancing beliefs away from themselves in each training session (anxiety, anger, sadness, and disgust). The order in which the four negative emotions is displayed will be randomized.
- *eAAMT-unpleasant emotions 1:4*: In the eAAMT-unpleasant emotions 1:4 condition, participants will also display a different unpleasant emotion in each training session (anxiety, anger, sadness, and disgust). However, there will be a response bias of 1:4 towards approach responses (i.e., on average for each dysfunctional belief, four functional beliefs are presented to strengthen the focus on resources such as functional beliefs and positive emotions).

#### Control interventions

- *Swipe control condition*: In the swipe control condition, participants will use the same app as in the experimental conditions. Here, the app will also provide participants with written instructions but without showing video clips. To approach and avoid the stimuli, participants will be asked to swipe stress-enhancing beliefs away from themselves and stress-reducing beliefs towards themselves on the smartphone screen. After swiping, the app will provide a respective zoom feedback and present a thumbs-up picture (see description of the experimental conditions). In the swipe control condition, the app will not be controlled by an experimenter. The participants will still be observed with an external video camera during the training to keep the conditions between experimental and control intervention equal.
- *Inactive control condition*: Participants randomized to the inactive control condition will not be invited to the lab and receive any training. They will receive links to the study questionnaires via email and fill them out by themselves.

### Measures

Table 1 gives an overview of all measures taken.

**Table 1:** Overview of measures per timepoint

**Variable**	**Instrument**	**Timepoint**^**a**^									
		**T0**	**T1**					**T2**			**T3**
			Pre	Tr1	Post	Tr2	Tr3	Pre	Tr4	Post	
Demographics	Self-devel	x									
Current and past psychotherapy	Self-devel	x									
**Feasibility outcomes**^**b**^											
Intervention usability	Self-devel									x	
App usability	SUS									x	
Acceptability	Self-devel									x	
**Clinical outcomes**											
*Psychometric measures*											
Perceived stress	PSS-10	x	x							x	x
Current stress level	Self-devel		x			x^b^	x^b^	x		x	x
Dysfunctional beliefs	DAS-A/H		x							x	x
Emotional state	Self-devel		x							x	x
Emotion regulation skills	ERSQ		x							x	x
Depressive symptoms	CES-D		x							x	x
*Performance measures*^*b*^											
Reaction time				x		x	x		x		
*N* of failed trials				x		x	x		x		
Felt intensity of emotion	Self-devel			x		x	x		x		
Rating of emotion display	Self-devel			x		x	x		x		
*Physiological measures*^*b*^											
Cortisol (saliva)	Saliva samples		x					x			
α-Amylase (saliva)											
Heart activity	Radar measurement		x		x			x		x	
Respiration											

Note: *Tr* Training, *self-devel* Self-developed questionnaire/item, *PSS-10* Perceived Stress Scale 10, *DAS-A* Dysfunctional Attitude Scale-Agreement, *DAS-H* Dysfunctional Attitude Scale-Helpfulness, *ERSQ* Emotion Regulation Skills Questionnaire, *CES-D* Center for Epidemiological Studies Depression Scale, *SUS* System Usability Scale. ^a^T0, screening; T1, assessment on the first day of training; T2, assessment on the last day of training; T3, assessment 1 week after training completion. At T1 and T2, measures will be taken before (pre) and after (post) the training. Tr1–4 are measures taken during the training after each trial. ^b^Measures are not taken in the inactive control group

#### Feasibility outcomes

With regard to feasibility, we aim to evaluate both the feasibility of the intervention and the possibility of a trial evaluating the efficacy of the intervention.

##### Feasibility of the intervention

Utilizing methods proposed in the context of process evaluation (e.g., Medical Research Council framework [72]), we will evaluate technical problems, adherence, usability, and acceptability. Technical problems will be evaluated by testing whether the proportion of incomplete usages of the intervention due to technical problems is above the a priori set criteria of 95%. Adherence will be evaluated by testing whether the proportion of participants completing the intervention as instructed is above the a priori set criteria of 50%. Usability of the intervention will be assessed with five self-constructed items rated on a 6-point Likert scale (0 to 5). They assess the following aspects of the intervention: comprehensibility of the instructions, viability of emotion expression at the beginning and end of the training, and degree of avoidance of stress-enhancing beliefs and degree of approach towards stress-reducing beliefs with the help of the intervention. In addition, the System Usability Scale (SUS) [73] will be administered in a subset of participants to assess the usability of the AAMT app. The scale consists of ten items measuring the global usability of technological systems on a 5-point Likert scale (1 = *strongly disagree* to 5 = *strongly agree*; exemplary item: “I thought the app was easy to use”). A total score can be obtained by reversely coding five of the ten items, summing them up and multiplying the sum by 2.5. The result is a score ranging from 0 to 100 with a higher score indicating better usability. Two additional items assess the general satisfaction with the app (1) on a 7-point Likert scale from *not at all satisfying* to *exceedingly satisfying* and (2) with school grades. The SUS is a reliable (Cronbach’s alpha = 0.91) and widely applicable measure [74]. Acceptability will be assessed with two self-constructed items. These items will be rated on a 6-point Likert scale (0 to 5). Participants will be asked to rate the helpfulness of the training for the modification of stress-related beliefs and whether they would recommend the training to a friend. The items will be analyzed separately. Additionally, participants will be asked in an open-ended format what they have learned from the training and whether they have general comments or recommendations.

##### Feasibility of the study design

We will collect information on the number of eligible participants and the percentage willing to participate in the study. Moreover, we will report the number of complete follow-up assessments and consider the study as feasible if at least 75% of the assessments will be completed.

#### Clinical outcomes

Primary outcome is the change in perceived stress level assessed by the German 10-item version of the PSS [66]. The PSS-10 assesses the unpredictability of, uncontrollability of, and state of being overwhelmed by daily life during the past week (exemplary item: “In the last week, how often have you been upset because of something that happened unexpectedly?”). The ten items are rated on a 5-point Likert scale (0 = *never* to 4 = *very often*). The ten items are summed up to receive a total score ranging between 0 and 40 with a higher total score representing a higher level of perceived stress. The PSS-10 has shown good reliability (Cronbach’s alpha = 0.84) and validity [66].

Secondary outcomes are symptoms of depression, emotion regulation skills, current stress level, stress-related (dys-)functional beliefs, emotional state, performance measures, and physiological stress measures.

*Symptoms of depression* will be assessed with the German version of the Center for Epidemiological Studies Depression Scale (CES-D; German version: [75]). The CES-D is a 20-item self-report questionnaire assessing the frequency of symptoms of depression in the general population during the last 7 days on a 4-point Likert scale (0 = *some of the time* to 3 = *most of the time*). The total score computed as the sum of all items can range between 0 and 60. Total scores higher than 22 are considered to indicate a clinically relevant depressive symptom severity [75]. The CES-D has shown good reliability (Cronbach’s alpha = 0.89–0.92) and validity in non-clinically depressed samples [76, 77].

*Emotion regulation skills* will be assessed with the Prolonged-State-Version of the Emotion Regulation Skills Questionnaire (ERSQ; German version: [78]). The self-report questionnaire measures the successful application of nine emotion regulation skills during the past week: *awareness* (e.g., “I paid attention to my feelings”), *sensations* (e.g., “My physical sensations were a good indication of how I was feeling”), *clarity* (e.g., “I was clear about what emotions I was experiencing”), *understanding* (e.g., “I was aware of why I felt the way I felt”), *modification* (e.g., “I was able to influence my negative feelings”), *acceptance* (e.g., “I accepted my emotions”), *tolerance* (e.g., “I felt I could tolerate my negative feelings”), *readiness to confront distressing situations* (e.g., “I did what I had planned, even if it made me feel uncomfortable or anxious”), and *self-support* (e.g., “I supported myself in emotionally distressing situations”). The 27 items are rated on a 5-point Likert scale (0 = *not at all* to 4 = *almost always*). In addition to the subscales assessing the specific emotion regulation skills, a general indicator of emotion regulation ability can be computed as the average score across all items (range = 0 to 4) with a higher score indicating higher emotion regulation skills. A number of studies demonstrated acceptable to good reliability (Cronbach’s alpha = 0.89–0.97) and validity of the questionnaire (e.g., [78–84]).

The *current stress level *will be assessed before each training session with a self-constructed short stress measure consisting of two items rated on an 11-point Likert scale (0 = *not at all* to 10 = *very much*): “Please indicate how stressed you feel at the moment” and “Please indicate how well you can cope with the stress at the moment.” The items will be analyzed separately.

To assess *stress-related (dys-)functional beliefs*, a total of ten items were selected from Rojas and colleagues’ [69] short version of the DAS. The DAS was developed as a self-report instrument to assess dysfunctional attitudes that are typically found in depressive patients. The ten items with the best fit for the stress context of the current study were selected by an expert group under supervision of MB: nine statements consisting of dysfunctional attitudes and one of a functional attitude. Then, two versions of the scale were created. The first version assesses the agreement with the ten DAS items on a 7-point Likert scale (DAS-A; 1 = *fully disagree* to 7 = *fully agree*) and the second version the perceived helpfulness of the beliefs (DAS-H; 1 = *not at all helpful* to 7 = *very helpful*). To obtain a total score, the functional-belief item is reversely coded, and the ten items are summed up. The total score in both versions ranges between 10 and 70. A higher score in the DAS-A represents higher agreement with dysfunctional stress-related beliefs, and a higher score in the DAS-H represents a higher helpfulness rating of dysfunctional beliefs.

To assess the *emotional state*, i.e., the intensity of emotions experienced during the last week, a team of experts in the assessment and regulation of emotions extended the German version of the Positive and Negative Affect Schedule [85] in a way that the following affective states are all assessed with one item: courage, gratitude, joy, pride, excitement, resolve, anger, anxiety, sadness, confidence, disgust, tranquility, love, happiness, relaxation, and content. Each item starts with the instruction “During the last week, I felt…” and invites participants to rate the intensity of the respective emotion during the past week on a 5-point Likert scale ranging from 0 = *not at all* to 4 = *very much*. The items will be analyzed separately.

#### Performance measures

The quality of participants’ emotion expression will be evaluated by the present investigator after each display of an emotion. The display in face, voice, and body will be rated on a 6-point Likert scale (0 = *no expression of the respective emotion* to 5 = *very strong expression of the respective emotion*). Moreover, participants will be asked to self-rate the felt intensity of the respective emotion after each stimulus on a visual analog scale presented on the smartphone (0 = *not at all* to 100 = *very much*). The smartphone app will also record the percentage of incorrect responses (expressing the wrong emotion in the eAAMT or swiping in the wrong direction in the swipe-based AAMT) as well as task completion time for all responses.

#### Physiological stress measures

To gain additional insights into the effects of the training on physiological stress systems, we will assess the concentration of hormonal stress markers as well as heart activity and respiration. Saliva samples for the assessment of cortisol and salivary alpha-amylase will be taken at the first and last training session before the training.

We will use Salivettes (Sarstedt, Nümbrecht, Germany) to collect the samples. The saliva samples will be analyzed at the Department of Health Psychology of the Friedrich-Alexander-Universität Erlangen-Nürnberg.

Heart activity and respiration will be recorded for 3.5 min in a subset of participants on day 1 and day 4 before and after the training. For this purpose, we will use a highly sensitive radar system, allowing for a contactless monitoring of the parameters of interest. For more details on the method, see [86]. Previous studies demonstrated the radar system to be a reliable and valid method for assessing heart activity and respiration in healthy individuals [87, 88]. We will be able to derive information on heart rate variability and breathing rate from these measurements.

#### Demographic information

We will also collect demographic data of the participants. Demographic data will include gender, age, education, current occupation, studies (degree, subject, semester), relationship status, psychotherapy (former and current), smoking, and medication.

### Procedures

Participants will be recruited via postings in social networks, the email newsletters of the Friedrich-Alexander-Universität Erlangen-Nürnberg, and flyers posted on notice boards in public places in Erlangen. The recruitment posts will provide interested individuals with a QR code and a link to the web site Unipark [89] where they will be informed about the study and will be given the opportunity to provide consent for participating in the first assessment. In case of informed consent, participants will be forwarded to the assessment of the inclusion and exclusion criteria. Individuals suitable for study inclusion will be randomized to one of the eight study conditions by the experimenters according to the randomization list. Neither participants nor experimenters will be blinded to the assignment.

During the training, the smartphone will record participants’ display of emotions with the integrated front camera. The recordings will be used as training material to train algorithms in automated emotion recognition (conducted in cooperation with the Chair of Embedded Intelligence for Healthcare and Wellbeing at the University of Augsburg and the Machine Learning and Data Analytics Lab at the Friedrich-Alexander-Universität Erlangen-Nürnberg). We will also record participants’ emotional expression with an external video camera and microphone. All of the video and sound recordings will only be used for training the algorithms and not be analyzed otherwise.

The clinical outcomes will be assessed at three time points via the web-based assessment tool Unipark [89]: directly before the training (T1), directly after the training (T2), and 1 week after training completion (T3). Feasibility outcomes will be assessed at T2. Additionally, participants in the intervention and swipe control groups will receive short daily questionnaires in paper–pencil format. Participants who do not fill out the T3 assessment will be reminded twice via email. In addition, participants who discontinue the intervention will be asked to answer the assessments anyway. Participants will be offered the opportunity to receive information about the results of the study via email.

Participants studying psychology will receive course credits for participation. Additionally, all participants enrolled in the study until May 2021 will have the chance of winning 500 € in a draw. From June 2021 onwards, participants will be offered an expense allowance of 20 € with psychology students having the choice between course credits and financial reimbursement. We undertook this change in procedure to compensate for COVID-19 associated problems with the recruitment.

### Ethical aspects

The study will be conducted following the Declaration of Helsinki and was approved by the German Psychological Society’s ethics committee (BerkingMatthias2020-09-10AM). All modifications of the study protocol will be amended at the ethics committee and documented in the trial register.

In the online screening questionnaire, we will collect demographic information (see above) as well as participant’s email addresses to be able to inform them about further proceedings in the study. Moreover, the online-platform Unipark logs IP addresses, date, time and content of the request, and device information (e.g., operating system and version, app version). Concerning data privacy, the platform Unipark complies with the European Union’s General Data Protection Regulation [90]. After the online screening, each participant will receive an ID number, and any identifying information (i.e., email address, name on the consent form) will be stored separately from the data collected in the study. A list in hard copy joining ID numbers and email addresses will only be accessible to the principal investigator. All data collected in the study (i.e., questionnaire, physiological, and smartphone data, video and sound recordings) will be stored on an external disk only accessible for persons directly involved in the study. The final data set including questionnaire, physiological, and smartphone data will be anonymized and made available in the Open Science Framework.

### Statistical analysis plan

Before conducting the analyses, we will test the preconditions and in case of violations use alternative methods. The level of significance will be set to *α* = 0.05. We will report *p*-values for all analyses.

Feasibility outcomes will be analyzed with descriptive statistics and independent *t*-tests to compare the results of the eAAMT conditions with the swipe control condition. To gain insight into the differences in clinical outcome measures between study conditions, we will compute pre-post effect sizes. In addition, we will conduct exploratory analyses using two-way mixed analyses of variance (ANOVAs) with treatment as between-subjects factor and time as within-subjects factor. Because omnibus tests fail to detect differences between single conditions if the majority of conditions do not differ, we plan to run pairwise comparisons comparing the single eAAMT conditions with the inactive control condition and the swipe control condition. In order to prevent alpha-error accumulation, we will first compare the eAAMT conditions to the inactive control condition. Only if the interaction effect in this comparison will be significant, we will also compare the eAAMT conditions to the swipe control condition. Moreover, as this is a pilot trial and we aim to evaluate if the training is effective in itself, we will conduct per protocol analyses. We will also run intention-to-treat analyses to determine the robustness of the results when considering missing data. All analyses will be conducted in R [91] and SPSS.

## Discussion

In this pilot study, we will evaluate the feasibility and explore the efficacy of a novel, emotion-based AAMT targeting stress-related beliefs in individuals with elevated perceived stress. Due to their important role in the emergence of perceived stress [20], stress-related beliefs are a promising starting point for interventions against stress. To our knowledge, this is the first study that employs an AAMT with stress-related beliefs as stimuli, thus targeting a construct directly implicated in the process of stress generation. Prior AAMT studies in the stress context that used emotional pictures as stimulus material mostly failed to find effects on stress measures [51, 54]. This demonstrates the need for a new way of designing AAMT interventions against stress, which our study addresses. Another novelty of our intervention is the use of emotions as approach/avoidance responses within an AAMT. Based on assumptions of the inferential account [35], we hypothesize that displaying highly valenced emotional responses within an AAMT will enhance the effects of currently performed AAMTs and be more effective in modifying approach-avoidance biases as well as reducing the particular psychopathology associated with such biases. In addition to their strong valence, the specific appraisals and behavioral tendencies associated with emotions [59] might be transferred to AAMT stimuli, thereby changing their evaluation in a more complex way. Therefore, in our study, participants will display emotional responses to push stress-related beliefs away from themselves and pull stress-reducing beliefs towards themselves. In order to provide an accessible and easy-to-disseminate training, we developed the eAAMT as app-based intervention. Since this is a completely new intervention, we decided to conduct a pilot study in preparation for a larger RCT. With the help of the feasibility evaluation, we will be able to gain insights into how the eAAMT can be implemented and is received by users. Moreover, we will be able to use the information on the feasibility of our study design to make further adaptations before the start of the RCT. By exploring the efficacy of the eAAMT, we will gain preliminary insights into its effects on clinical outcomes and, hence, therapeutic potential. We hypothesize that the eAAMT will be more effective in reducing perceived stress as well as the extent of agreement with dysfunctional beliefs and symptoms of depression than a swipe-based AAMT. In addition to exploring this hypothesis, we will explore whether the display of the emotions anxiety, anger, sadness, or disgust will be particularly effective and whether a bias towards approach responses within the eAAMT increases its efficacy. When we developed the protocol, we decided to use an inactive control condition as one of the comparators. This allows us to answer the question whether the eAAMT is helpful at all. As second comparator, we chose to use the swipe-based AAMT which is currently the most common AAMT variant on the smartphone [39–41, 52, 53]. The comparison with a swipe-based AAMT allows us to investigate whether the use of emotions in the AAMT provides additional benefit.

The current study has several limitations. First, we will use standardized stress-related beliefs that may not be relevant for every participant. However, we aim to develop an app that can be disseminated on a large scale and used by everyone and thus decided to standardize the material. Second, the eAAMT will be performed as a Wizard-of-Oz paradigm [92] in which the investigator assesses the emotional response as long as the algorithms are not able to do this (however, in contrast to a real Wizard-of-Oz paradigm, participants are informed that the investigator assesses the emotional response). The presence of an investigator, however, might have an impact on participants’ performance [93], especially if they are socially anxious, and therefore influence the efficacy of the app in reducing perceived stress. Thus, future studies should compare the efficacy of an investigator-based eAAMT with the automated version of the intervention. Third, our target sample size is very small. It is sufficient, however, to test the feasibility of the intervention and study design and explore its therapeutic potential. If the results of the intended study are promising, future studies should investigate its efficacy in a bigger sample. Fourth, we will not be able to draw conclusions about the long-term efficacy of the intervention. Thus, in case of evidence for short-term effects, future studies should include long-term follow-up assessments. Fifth, the compensation for study participation is not equal over the course of the study. Psychology students receive course credit at all times. Non-psychology students, on the other hand, did not receive compensation at the beginning of the study, but it was introduced during the course of the study. The presence of a compensation might reduce intrinsic motivation to participate in the intervention and thus affect the outcomes. However, we believe that course credit or a compensation of 20 € is not enough to be the only source of motivation to participate in such a study. Nevertheless, future studies should keep compensation equal. Finally, recruitment of the pilot trial already had started by the time this study protocol was written. This is not in line with good scientific practice which requires the publishing of study protocols before the start of the trial to increase transparency. However, to ensure transparency, this pilot study has been preregistered. We decided to additionally publish the study protocol because it allows us to describe the complex design and novel intervention in more detail.

All these limitations aside, if shown to be effective, the eAAMT has great potential to improve healthcare as it is an easy-to-deliver and engaging intervention. Moreover, approach-avoidance biases are implicated in many mental disorders [33]. Hence, increasing the efficacy of currently employed interventions might improve treatment in various domains of psychopathology. In addition, if, as endeavored in our study, the emotion recognition is automated with the help of machine learning methods and implemented in future interventions, such new, innovative approaches open up many possibilities to further reduce the burden on the healthcare system — important assets in order to address “the health epidemic of the twenty-first century” [1].

### Trial status

We conducted a preliminary feasibility trial in June and July 2020 with *N* = 16 participants. After minor adaptations to the experimental setting, recruitment for the pilot trial started in July 2020 and is currently ongoing.

## Data Availability

The final data set including questionnaire, physiological, and smartphone data will be anonymized and made available in the Open Science Framework.

## Abbreviations

AAMT: Approach-avoidance modification training
CBM: Cognitive bias modification
CES-D: Center for Epidemiological Studies Depression Scale
DAS: Dysfunctional Attitude Scale
eAAMT: Emotion-based approach-avoidance-modification training
ERSQ: Emotion Regulation Skills Questionnaire
PSS: Perceived Stress Scale
SUS: System Usability Scale
